# Inhibition of Telomerase Activity by Oleanane Triterpenoid CDDO-Me in Pancreatic Cancer Cells is ROS-Dependent

**DOI:** 10.3390/molecules18033250

**Published:** 2013-03-13

**Authors:** Dorrah Deeb, Xiaohua Gao, Yongbo Liu, Nadimpalli R. S. Varma, Ali S. Arbab, Subhash C. Gautam

**Affiliations:** 1 Department of General Surgery, Henry Ford Health System, Detroit, MI 48202, USA; E-Mails: ddeeb1@hfhs.org (D.D.); tgao1@hfhs.org (X.G.); bliu1@hfhs.org (Y.L.); 2 Department of Radiology Research, Henry Ford Health System, Detroit, MI 48202, USA; E-Mails: ravin@rad.hfh.edu (N.R.S.V.); sali1@hfhs.org (A.S.A.)

**Keywords:** pancreatic cancer, CDDO-Me, ROS, hTERT, telomerase activity

## Abstract

Methyl-2-cyano-3,12-dioxooleana-1,9(11)-dien-28-oate (CDDO-Me) is a synthetic derivative of oleanolic acid, a triterpene, with apoptosis-inducing activity in a wide range of cancer cells. Induction of apoptosis by CDDO-Me is associated with the generation of reactive oxygen species (ROS) and inhibition of telomerase activity. In the present study, we investigated the role of ROS in inhibition of telomerase by CDDO-me. Treatment of MiaPaCa-2 and Panc-1 pancreatic cancer cell lines with CDDO-Me induced the production of hydrogen peroxide and superoxide anions and inhibited the telomerase activity. Pretreatment of cells with N-acetylcycsteine, a general purpose antioxidant or overexpression of glutathione peroxidase (GPx) or superoxide dismutase-1 (SOD-1) blocked the telomerase inhibitory activity of CDDO-Me. Furthermore, blocking ROS generation also prevented the inhibition of hTERT gene expression, hTERT protein production and expression of a number of hTERT–regulatory proteins by CDDO-Me (e.g., c-Myc, Sp1, NF-κB and p-Akt). Data also showed that Akt plays an important role in the activation of telomerase activity. Together, these data suggest that inhibition of telomerase activity by CDDO-Me is mediated through a ROS-dependent mechanism; however, more work is needed to fully understand the role of ROS in down-regulation of hTERT gene and hTERT-regulatory proteins by CDDO-Me.

## 1. Introduction

2-Cyano-3,12-dioxooleana-1,9(11)-dien-28-oic acid (CDDO) and its C-28 methyl ester (CDDO-Me) and C-28 imidazole (CDDO-Im) are synthetic derivatives of oleanolic acid (OA) with several-fold greater anti-inflammatory activity than OA [1]. CDDOs have shown potent antiproliferative and apoptosis-inducing activity in diverse types of tumor cell lines, including leukemia, multiple myeloma, osteosarcoma, breast, brain, prostate and lung cancer cells [2,3,4,5,6]. In previous studies, we have demonstrated that the antiproliferative and apoptosis inducing activity of CDDO-Me in prostate and pancreatic cancer cell lines is mediated through the inhibition of anti-apoptotic (prosurvival) Akt/NF-κB/mTOR signaling pathways [7,8]. These studies also revealed that generation of reactive oxygen species (ROS) is involved in the antiproliferative and apoptosis-inducing activity of CDDO-Me [9,10].

Recently we showed that telomerase, the reverse transcriptase that elongates telomeres, is a potential target of CDDO-Me cancer cells [11,12]. Telomeres are nucleoprotein structures present at the end of chromosomes that maintain chromosome stability by preventing end-to-end fusion and prevent chromosomal rearrangement [13]. The human telomerase complex consists of telomerase reverse transcriptase (hTERT), telomerase RNA (TERC), telomerase associated protein-1 (TEP-1), hsp90 and p23 [14,15,16]. The telomere length is progressively shortened due to gradual loss of telomeric DNA sequence (TTAGGG) during each cell division and shortening of telomeres beyond a critical threshold leads to the replicative senescence or apoptosis [17,18]. Telomerase maintains telomere length by adding the hexameric DNA repeats (TTAGGG) to the 3' flanking end of DNA strands in telomeres. 

Deregulated telomerase activity is associated with the promotion of tumorigenesis and neoplastic growth of cancers [19,20]. Approximately 90% of human cancers exhibit reactivation of telomerase activity [21], therefore, telomerase is an attractive target for developing novel anticancer therapeutics. Indeed, we have recently shown that inhibition of cell proliferation and induction of apoptosis by CDDO-Me in pancreatic cancer cells is associated with the repression of hTERT expression, the gene that codes for telomerase, and telomerase activity [11]. Since ROS is involved in the antiproliferative and apoptosis-inducing activity of CDDO-Me, in the present study we investigated the role of ROS in inhibition of telomerase activity in pancreatic cancer cells by CDDO-Me. 

## 2. Results and Discussion

### 2.1. CDDO-Me Induces ROS Generation in Pancreatic Cancer Cells

**Figure 1:**
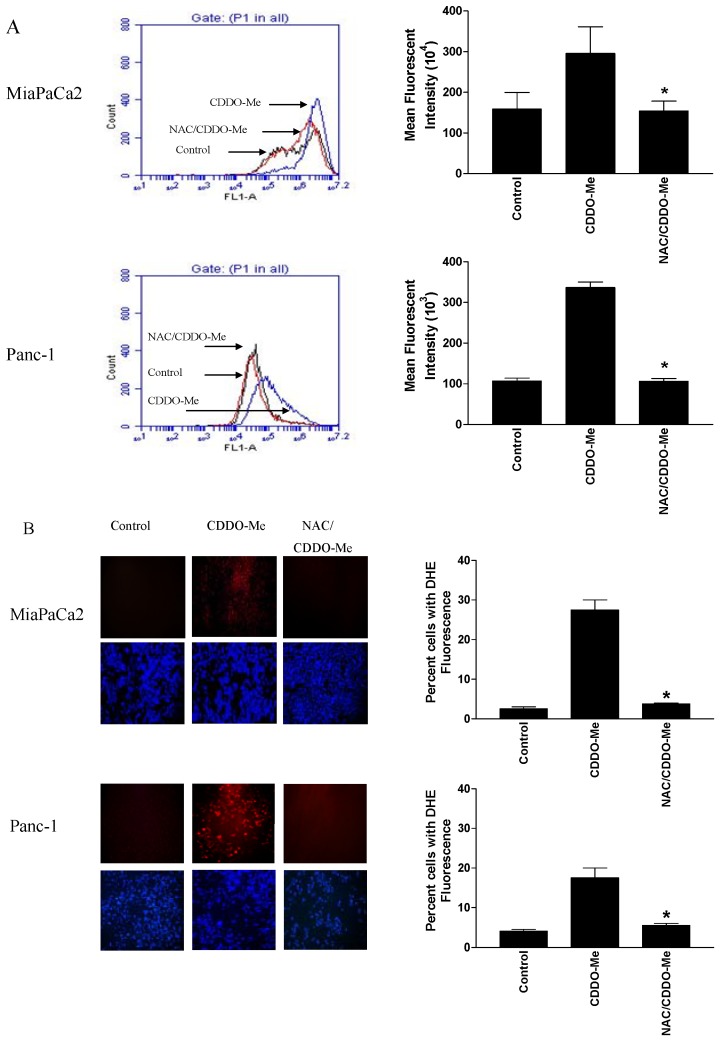
CDDO-Me induces ROS production in pancreatic cancer cells and NAC blocks it. (**A**) MiaPaCa-2 cells and Panc-1 cells pretreated or not with NAC (3 mM) for 2 h were treated with CDDO-Me (1.25 μM) for 2 h. Cells were then reacted with 5 μM H_2_DCFDA for 30 min at 37 °C and DCF fluorescence was measured by flow cytometry; (**B**) For DHE staining, after treatment with CDDO-Me as described above, MiaPaCa-2 cells and Panc-1 cells were reacted with DHE for 30 min. Cells were counter stained with Hoechst dye and observed under fluorescent microscope (200× magnification). NAC completely blocked the DHE fluorescence induced by CDDO-Me. * *p* < 0.05.

To establish the role of ROS in inhibition of hTERT telomerase activity by CDDO-Me we first measured the generation of ROS in MiaPaCa-2 and Panc-1 pancreatic cancer cells. The generation of ROS by CDDO-Me was measured by using H_2_DCFDA and DHE fluorescent probes that detect H_2_O_2_ and O_2_^●−^, respectively. As shown in Figure 1A, treatment of both cell lines with 1.25 μM CDDO-Me for 2 h resulted in two–fold (MiaPaCa-2) and three-fold (Panc-1) increase in DCF fluorescence intensity as measured by flow cytometry. Pretreatment of cells with antioxidant NAC completely prevented the increase in DCF fluorescence intensity by CDDO-Me in both cell lines.

DHE staining of cells was measured by fluorescent microscopy. As shown in Figure 1B, there was minimal staining with DHE in untreated control cells (both cell lines). On the other hand, treatment with CDDO-Me (1.25 μM) for 2 h significantly increased DHE staining in both cell lines (30% and 17%, respectively). Pretreatment of cells with NAC completely blocked the CDDO-Me-induced DHE staining in both cell lines. 

### 2.2. NAC Blocks the Antiproliferative and Apoptosis-Inducing Activity of CDDO-Me

**Figure 2:**
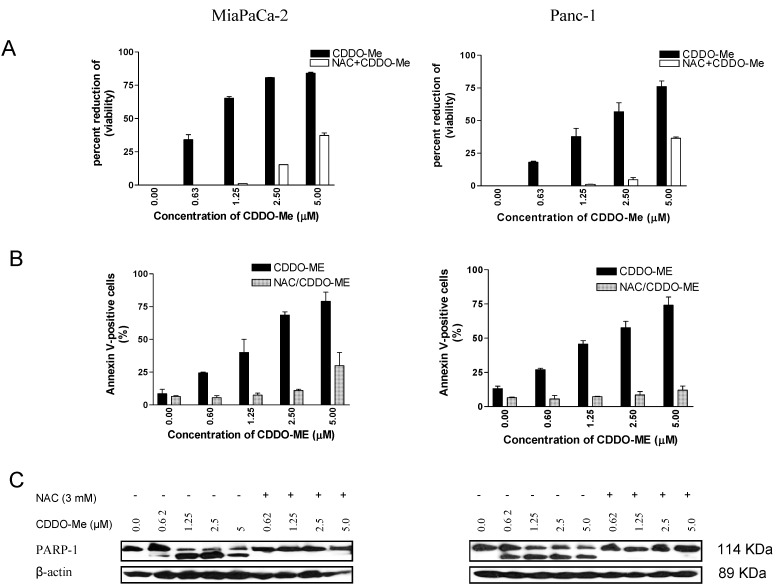
NAC blocks inhibition of pancreatic cancer cell proliferation by CDDO-Me. (**A**) 1 × 10^4^ MiaPaCa-2 or Panc-1 cells pretreated or not with NAC (3 mM) for 2 h were treated with CDDO-Me at concentrations ranging from 0 to 5 µM for 72 h in triplicate in 96-well microtiter plates. Cell viability was measured by MTS assay using CellTiter AQueous assay system from Promega. Data are presented as percent reduction in viability obtained in three independent experiments; (**B**) CDDO-Me induces apoptosis in pancreatic cancer cells and NAC blocks it. For annexin V-FITC binding, MiaPaCa-2 and Panc-1 cells were treated or not with NAC (3 mM) for 2 h prior to treating with CDDO-Me at concentrations of 0 to 5 µM for 24 h. Cells were then reacted with 5 μL of annexin V-FITC + PI for 30 min at room temperature. The percentage of annexin V-FITC positive tumor cells was determined by flow cytometry; (**C**) PARP-1 cleavage in MiaPaCa-2 cells and Panc-1 cells treated or not with NAC (3 mM) for 2 h prior to treatment with CDDO-Me for 24 h was analyzed by Western blotting. Each experiment was repeated two times.

To determine whether generation of intracellular ROS plays a role in the antiproliferative activity of CDDO-Me, MiaPaCa-2 and Panc-1 cells were pre-treated or not with NAC for 2 h before treating with CDDO-Me (0.625 to 5 µM) for 72 h and viability of cell cultures was determined by MTS assay. As shown in Figure 2A, CDDO-Me inhibited the viability of both cell lines in a dose-dependent manner. 

In MiaPaCa-2 cells, significant reduction in viability was evident at 0.625 µM CDDO-Me (31%) which increased to 64%, 76% and 81% reduction in viability at 1.25, 2.5 and 5 µM CDDO-Me, respectively (*p* < 0.05). Measurable reduction in viability (13%) was observed in Panc-1 cells at 0.625 µM CDDO-Me, which further increased from 38% to 77% reduction at 1.25–5 µM CDDO-Me. In contrast, pre-treatment with NAC almost completely blocked the antiproliferative effect of CDDO-Me in both cell lines up to a concentration of 2.5 µM. At 5 µM CDDO-Me, protection provided by NAC was significantly reduced (~50%).

To investigate whether CDDO-Me induces apoptosis, MiaPaCa-2 and Panc-1 cells treated with CDDO-Me for 24 h were reacted with annexin V-FITC and analyzed by flow cytometry. As shown in Figure 2B, treatment with CDDO-Me increased annexin V-FITC binding in both cell lines dose-dependently (MiaPaCa-2, 6% to 76%; Panc-1, 10% to 73% at 0 and 5 µM CDDO-Me. In contrast, pretreatment with NAC dramatically blocked the CDDO-Me-induced binding of annexin V-FITC at all concentrations in both cell lines.

The effect of NAC on the cleavage of PARP-1 by CDDO-Me was also examined. Figure 2C clearly shows the cleavage of PARP-1 by CDDO-Me as demonstrated by the presence of 89 kDa cleaved PARP-1 fragment at concentrations of 1.25 to 5 µM in both cell lines. On the other hand, pretreatment with NAC blocked the cleavage of PARP-1 by CDDO-Me. Taken together, these data indicated that ROS generation plays a role in the antiproliferative and apoptosis-inducing activity of CDDO-Me.

### 2.3. Antioxidants Block Inhibition of hTERT Telomerase Activity by CDDO-Me

**Figure 3:**
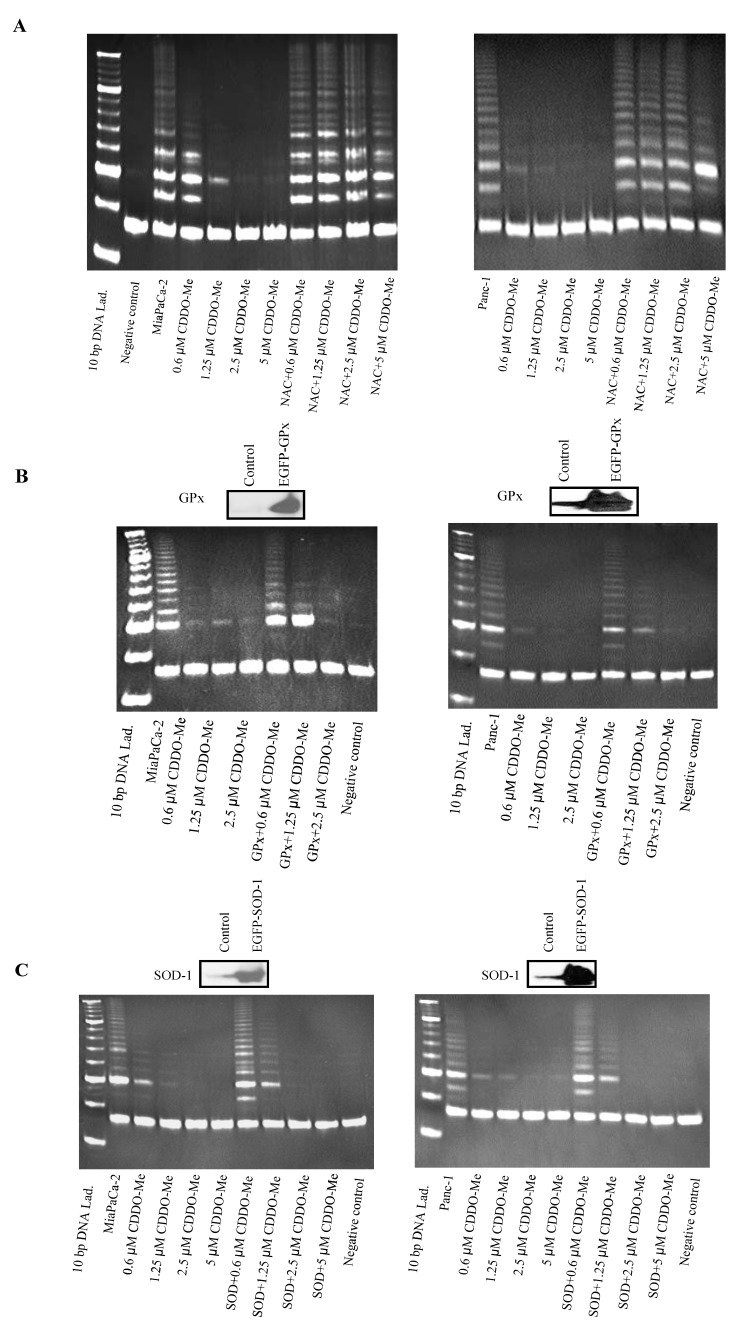
CDDO-Me inhibits telomerase activity and antioxidants block it. (**A**) Effect of NAC. MiaPaCa-2 and Panc-1 cells pretreated or not with NAC (3 mM) for 2 h were treated with CDDO-Me (0–10 μM) for 48 h and telomerase activity of cell extracts was measured by TRAP assay as described in Materials and Methods. DNA laddering patterns under different treatment conditions are shown. Experiments were repeated two times. NC, negative control (no cell extract); (**B**,**C**) Effect of overexpression of antioxidant enzymes GPx and SOD-1. MiaPaCa-2 and Panc-1 cells were transfected with GPx or SOD-1 expression plasmids using LipofectAMINE Plus reagent for 48 h. Overexpression of enzymes was confirmed by immunoblotting and cells were then treated with CDDO-Me at 0.625–5 µM for 48 h and telomerase activity was measured by TRAP assay.

We recently showed that CDDO-Me inhibits hTERT telomerase activity in pancreatic cancer cells [11]. Since ROS generation is involved in the antiproliferative and proapoptotic activity of CDDO-Me we next evaluated whether it also has a role in the inhibition of hTERT telomerase activity by CDDO-Me. First, the effect of NAC on inhibition of telomerase activity by CDDO-Me in MiaPaCa-2 and Panc-1 cells was evaluated. For this, tumor cells were treated with CDDO-Me (0.625–5 μM) for 48 h and cells were extracted in CHAP lysis buffer. The telomerase activity of extracts was measured using the PCR-based TRAP assay method. As shown in Figure 3A, there was ~40% reduction in the telomerase activity in both cell lines treated with 0.625 μM CDDO-Me. The telomerase activity was sharply to completely inhibited in cells treated with CDDO-Me at concentrations of 1.25 to 5 μM as identified by the loss of DNA laddering in both cell lines. In contrast, pretreatment with NAC almost completely blocked the inhibition of telomerase activity by CDDO-Me in both cell lines.

To further confirm the role of ROS in inhibition of telomerase activity by CDDO-Me, we examined the effect of CDDO-Me on the telomerase activity in MiaPaCa-2 and Panc-1 cells that were transduced to overexpress antioxidant enzymes glutathione peroxidase (GPx) or superoxide dismutase-1 (SOD-1). As shown in Figure 3B, overexpression of GPx and SOD-1 protected both cell lines from the inhibition of telomerase activity by CDDO-Me at 0.625 and 1.25 μM CDDO-Me. There was some protection of telomerase activity at 2.5 μM CDDO-Me in cells overexpressing GPx but not SOD-1 and there was no protection against 5 μM CDDO-Me in cells overexpressing GPx or SOD-1. Together, these data indicated that antioxidants protect tumor cells from the inhibition of hTERT telomerase activity by CDDO-Me.

### 2.4. NAC Blocks the Inhibition of hTERT and hTERT Regulatory Proteins by CDDO-Me

**Figure 4:**
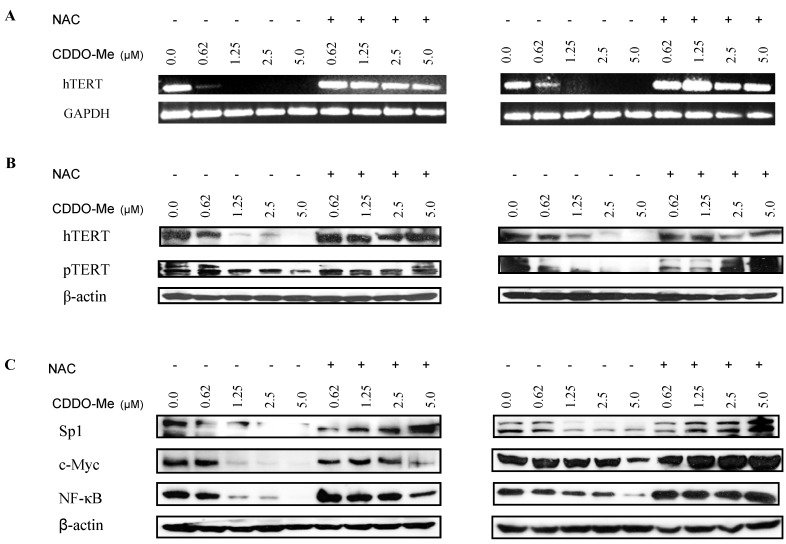
NAC blocks the inhibition of hTERT expression and hTERT related transcription factors by CDDO-Me. (**A)** Effect on hTERT gene expression. Mia PaCa-2 and Panc-1 cells pretreated or not with NAC (3 mM) were treated with CDDO-Me (0–5 μM) for 48 h and total cellular RNA was prepared using TRI-zole reagent. 1 μg of cellular RNA was reverse transcribed using oligo-dt primer and high fidelity reverse transcriptase. 1 μL of cDNA was amplified using hTERT or GAPDH primers. Amplified products were separated on 2% DNA agarose gel. Gels were stained with ethidium bromide and amplified DNA fragments were identified by base pair sizes; (**B**) Effect on hTERT protein. Mia PaCa-2 and Panc-1 cells were treated with CDDO-Me (0–5 μM) for 48 h as above and cell lysates were analyzed for hTERT and p-hTERT protein by western blotting; (**C**) Effect on hTERT transcription factors. MiaPaCa-2 and Panc-1 cells were treated with CDDO-Me (0–5 μM) for 48 h as in **A** and cell lysates were analyzed for c-Myc, Sp1, and NF-κB by Western blotting. Each experiment was repeated at least two times.

ROS has been implicated in gene expression and activation of transcription factors. We have previously shown that CDDO-Me inhibits hTERT gene expression as well as transcription factors and signaling proteins that regulate hTERT gene expression [11]. Therefore, we next tested whether ROS was involved in the repression of hTERT gene and hTERT regulatory proteins by CDDO-Me. We first evaluated the effect of antioxidant NAC on the inhibitory effect of CDDO-Me on expression of hTERT and hTERT associated proteins. The expression of hTERT mRNA and hTERT protein was analyzed by RT-PCR and Western blotting, respectively. Treatment with CDDO-Me completely inhibited hTERT mRNA in both cell lines at 1.25 to 5 μM CDDO-Me, which was completely blocked when cells were pretreated with NAC before applying CDDO-Me (Figure 4A). Similarly, NAC also protected cells from the inhibition of total hTERT and p-hTERT (ser^826^) protein by CDDO-Me (Figure 4B).

Transcription of hTERT gene is regulated by the transcription factor c-Myc, Sp1 and NF-κB. Therefore, we assessed whether ROS is involved in down-regulation of these transcription factors by CDDO-Me. Treatment with CDDO-Me (0.625 to 5 μM) for 48 h significantly reduced the levels of c-Myc, Sp1 and NF-κB in a dose-related manner in both cell lines (Figure 4C). In contrast, pretreatment with NAC protected cells from the inhibition of these transcription factors by CDDO-Me, suggesting that inhibition of hTERT transcription factors by CDDO-Me is mediated through a ROS-dependent pathway.

### 2.5. Akt Regulates hTERT Telomerase Activity

**Figure 5:**
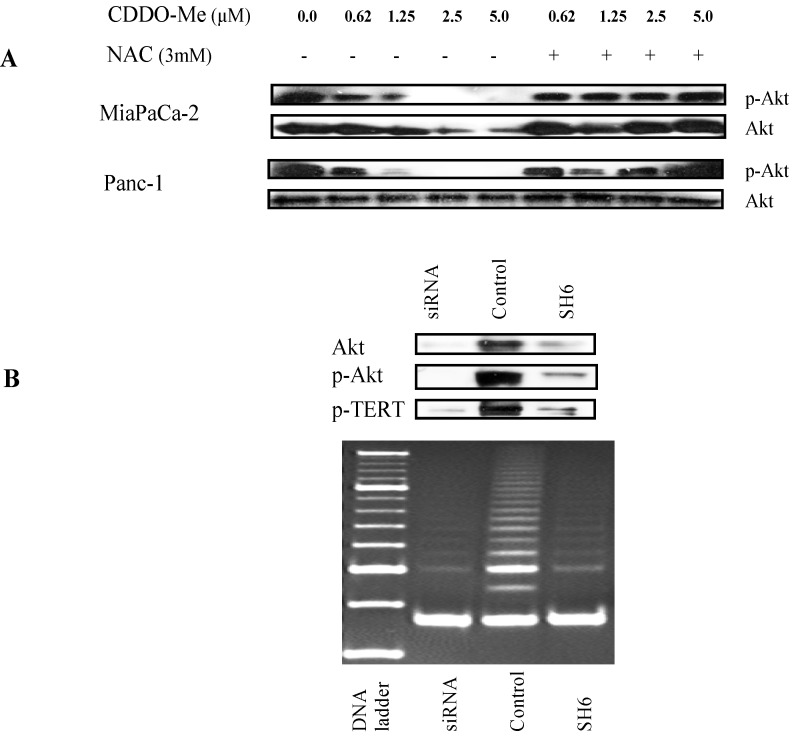
NAC blocks inhibition of p-Ak by CDDO-Me and inhibition of p-Akt inhibits telomerase activity of hTERT. (**A**) MiaPaCa-2 and Panc-1 cells were pretreated with NAC (3 mM) for 2 h followed by treatment with CDDO-Me (0–5 μM) for 48 h. Cell lysates were analyzed for p-Akt and total Akt by western blotting; (**B**) Inhibition of p-Akt inhibits telomerase activity. Panc-1 cells were transfected with siRNA-Akt or treated with Akt inhibitor SH6 before treating with CDDO-Me (0–5 μM) for 48 h. Cell lysates were analyzed for Akt, p-Akt and p-TERT by western blotting and telomerase activity was measured by TRAP assay.

Post-translationally, hTERT is regulated by serine kinase Akt. Phosphorylation of hTERT on ser^227^ and ser^824^ by Akt is essential for hTERT activation and nuclear translocation. The effect of NAC on inhibition of p-Akt by CDDO-Me was evaluated. As shown in Figure 5A, CDDO-Me inhibited p-Akt in both cell lines in a dose-related manner with complete inhibition at 2.5–5 μM CDDO-Me. There was little change in basal Akt in Panc-1 cells but it was significantly reduced, especially at 2.5–5 μM CDDO-Me in MiaPaCa-2 cells. Pretreatment with NAC reversed the effect of CDDO-Me on p-Akt, suggesting that ROS is involved in the inhibition of phosphorylation of Akt by CDDO-Me.

We also analyzed the effect of selectively knocking down Akt on hTERT telomerase activity. For this purpose, Panc-1 cells were transfected with siRNA-Akt or treated with Akt specific inhibitor SH6. As can be seen in Figure 5B, knocking-down Akt with siRNA-Akt or SH6 inhibitor not only inhibited p-Akt but also p-hTERT (western blot). Dephosphorylation of hTERT subsequently resulted in the inhibition of telomerase activity. This result highlights that inhibition of Akt by CDDO-Me plays a pivotal role in inhibition of hTERT telomerase activity by CDDO-Me.

### 2.6. Discussion

Deregulated telomerase activity is associated with the promotion of tumorigenesis and neoplastic growth of cancers and telomerase is reactivated in more than 90% of all cancers [19,20,21]. Thus, cancer selective reactivation of telomerase provides an opportunity to develop new anticancer therapeutics selectively targeting the telomerase. The suppression of telomerase in cancer cells leads to cellular senescence and induction of apoptosis [22]. Indeed, inhibition of cell proliferation and induction of apoptosis in pancreatic cancer cells by CDDO-Me was associated with the inhibition of telomerase activity, suggesting that suppression of telomerase activity by CDDO-Me is at least partly responsible for the anticancer activity of CDDO-Me. These results are in agreement with other reports showing that inhibition of hTERT telomerase activity is necessary for the antiproliferative and apoptosis-inducing activity of compounds such as genistein, sulforaphane, amooranin and curcumin [23,24].

Present studies were aimed at determining the role of reactive oxygen species in the telomerase inhibitory activity of CDDO-Me in pancreatic cancer cells. ROS are generated intracellularly as byproducts of normal aerobic metabolism or as second messengers in various signal transduction pathways or in response to environmental stress [25,26]. Depending upon the concentration, ROS elicit a wide spectrum of biological responses ranging from mitogenic to proliferative effects at low concentration to macromolecular damage and cell death at high concentrations [27]. Further, generation of ROS is part of the mechanism by which most chemotherapeutic agents and ionizing radiation kill tumor cells [28,29]. CDDO-Me generated ROS in cancer cells as CDDO-Me caused the oxidation of H_2_DCFDA and DHE, indicating production of hydrogen peroxide and superoxide anion, respectively. The generation of ROS by CDDO-Me was blocked by NAC, a small molecule general antioxidant. Similarly, NAC also blocked the inhibition of cell proliferation and induction of apoptosis by CDDO-Me, indicating participation of ROS in the anticancer activity of CDDO-Me. These findings are consistent with the results of previous studies showing participation of ROS in killing of leukemia and pancreatic cancer cells by CDDO-Me [30].

The effect of ROS on hTERT telomerase activity is controversial. ROS has been shown to inhibit telomerase activity by natural products in some studies, but was shown to increase it in others [31,32,33,34]. In our studies, pretreatment with NAC prevented the inhibition of telomerase activity by CDDO-Me. In addition, overexpression of antioxidant enzymes GPx and SOD-1 also mitigated the telomerase inhibitory activity of CDDO-Me. Together, these data suggested that ROS plays a role in the antitelomerase activity of CDDO-Me.

The inhibition of telomerase activity by CDDO-Me might result from inhibition of hTERT gene expression, hTERT protein production or hTERT activation by Akt. Indeed, analysis of hTERT mRNA by RT-PCR and hTERT protein by western blotting showed that CDDO-Me attenuates hTERT mRNA and phosphorylated hTERT protein. NAC blocked these effects of CDDO-Me, implying that ROS generated by CDDO-Me plays a role in the antitelomerase activity of CDDO-Me. 

hTERT is regulated both transcriptionally and post-translationally. The hTERT core promoter contains binding sites for transcription factors such as c-Myc, Sp1, NF-κB and STAT-3 and Sp1 cooperates with c-Myc to enhance hTERT expression [31,35,36]. Sp transcription factors regulate the transcription of tumorigenesis related genes (e.g., cyclin D1, survivin and VEGF) and are targets of CDDO-Me like compounds such as betulinic acid and its synthetic derivatives [37,38,39]. Therefore, it was not surprising that CDDO-Me inhibited Sp1 and other transcription factors that control hTERT expression. Post-translationally, phosphorylation of hTERT on Ser^227^ and Ser^824^ by Akt is essential for activation of its telomerase activity [40,41]. CDDO-Me inhibited the phosphorylation of Akt which in turn prevented phosphorylation of hTERT and activation of its telomerase activity. Furthermore, the inhibition of Akt and NF-κB by CDDO-Me could also impair nuclear accumulation of hTERT since hTERT associates with both Akt and NF-κB for nuclear translocation [40]. Here also, pretreatment with NAC mitigated the inhibitory effect of CDDO-Me on all of the proteins involved in hTERT expression and activation, indicating that ROS may have a role in inhibition of hTERT-regulatory proteins by CDDO-Me. Although the role of ROS in cell growth regulation is complex, free radicals have been shown to activate pro-survival pathways including MAP kinases as well as Akt and NF-κB signaling pathways [42]. What exactly is the role of ROS in inhibition of hTERT expression, its regulation and telomerase activity is unclear at present? Clearly more work is needed to precisely define the role of ROS in inhibition of hTERT telomerase complex to maximize the therapeutic potential of CDDO-Me for targeting telomerase in cancer cells.

## 3. Experimental

### 3.1. Reagents

CDDO-Me was obtained from the National Cancer Institute (Bethesda, MD, USA) through the Rapid Access to Intervention Development Program. A 100 mM stock solution of CDDO-Me was prepared in DMSO, which was subsequently diluted in tissue culture medium to obtain the working concentrations. Antibodies against PARP-1, Akt, p-Akt (ser^473^), NF-κΒ (p65), Sp1, c-Myc, SOD-1, GPx and β-actin were purchased from Santa Cruz Biotechnology, Inc. (Santa Cruz, CA, USA). Anti-hTERT and p-TERT (Ser^824^) antibodies were obtained from-Abcam Inc. (Cambridge, MA, USA). CellTiter 96® AQueous One Solution Proliferation Assay System was from Promega (Madison, WI, USA). Annexin V-FITC apoptosis detection kit II was obtained from BD Pharmingen (San Diego, CA, USA) and TRAPeze telomerase detection kit was purchased from Millipore (Millipore, Temecula, CA, USA). H_2_DCF-DA and DHE fluorescent probes were from Molecular Probes (Eugene, OR, USA). 

### 3.2. Cell Lines

Human pancreatic cancer cell lines Mia-PaCa-2 and Panc-1 were obtained from the American Type Culture Collection (ATCC, Rockville, MD, USA). Cell lines were cultured at 37 °C in a humidified atmosphere consisting of 5% CO_2_ and 95% air and maintained by subculturing cells twice a week. 

### 3.3. Measurement of Cell Viability

1 × 10^4^ cells in 100 μL of cell culture medium were seeded into each well of a 96-well plate. After incubation for 24 h, cells were treated with CDDO-Me for additional 72 h. Cell viability was then determined by the colorimetric MTS assay using CellTiter 96 AQueous One Solution Proliferation Assay System from Promega. After incubation for 2 h at 37 °C, optical density was measured at 490 nm using a microplate reader. 

### 3.4. Apoptosis Assay

Apoptosis was assessed by the binding of annexin V-FITC to phosphotidylserine, which is externalized to the outer leaflet of the plasma membrane early during induction of apoptosis. Briefly, untreated cells and cells treated with CDDO-Me for 24 h were resuspended in the binding buffer provided in the annexin V-FITC apoptosis detection kit II (BD Biosciences, San Diego, CA, USA) and reacted with 5 μL of annexin V-FITC reagent and 5 μL of propidium iodide (PI) for 30 min at room temperature in the dark. Stained cells were analyzed by flow cytometry using Accuri C6 flow cytometer (Accuri Cytometers Inc. Ann Arbor, MI, USA). The induction of apoptosis by CDDO-Me was confirmed from the cleavage of PARP-1 by western blotting.

### 3.5. Measurement of ROS

H_2_DCF-DA and DHE fluorescent probes were used to measure intracellular generation of hydrogen peroxide (H_2_O_2_) and superoxide anions (O_2_^●−^) respectively. Briefly, 1 × 10^6^ Mia-PaCa-2 or Panc-1 were plated in 6-well plates and allowed to attach overnight. Cells were treated or not with CDDO-Me and then reacted with 5 µM of H_2_DCF-DA or 2 µM of DHE for 30 min at 37 °C. Cells were collected by trypsinization and DCF-DA fluorescence was analyzed by flow cytometry. For DHE fluorescence, cells were counterstained with Hoechst dye and fluorescence emission was detected by a Leica fluorescent microscope (Heerburg, Switzerland). 

### 3.6. Measurement of hTERT Expression

The effect of CDDO-Me on hTERT expression was measured by analyzing hTERT mRNA and hTERT protein. For hTERT mRNA, total cellular RNA was extracted with TRI-zol reagent (GIBCO) according to the manufacturer’s recommendation. 1 μg of RNA was then reverse transcribed by oligo-dt primer and high fidelity reverse transcriptase (Roche Diagnostics, Mannheim, Germany) to generate cDNAs. One μL of cDNA was used as the template for polymerase chain reaction (PCR) using hTERT primers: upper, 5'-TGTTTCTGGATTTGCAGGTG-3', and lower, 5'-GTTCTTGGCTTTCAGGA TGG-3'; and GAPDH primers: upper, 5'-TCC CTC AAG, ATT, GTC AGC AA-3', and lower, 5'-AGA TCC ACA ACG GAT ACA TT-3'. The PCR conditions used were 33 cycles of denaturation (95 °C for 1 min), annealing (62 °C for 30 s), and polymerization (72 °C for 1 min). The PCR products were separated on 2% agarose gel electrophoresis and visualized by ethidium bromide staining. Gels were photographed and band densities were analyzed using the NIH/Scion image analysis software. The hTERT primers amplified a DNA fragment of 200 bp and the DNA fragment size amplified by GAPDH primers was 173 bp.

### 3.7. Telomerase Activity Assay

The telomerase activity in cell extracts was assessed by the PCR-based telomeric repeat amplification protocol (TRAP) using TRAPeze gel-based telomerase detection kit (Millipore, Temecula, CA, USA) following the instructions provided in the kit. Briefly, cells were extracted in CHAP lysis buffer on ice for 30 min. Two μL (100 ng) of cell extract was added to the TRAP reaction mixture containing dNTPs, TS primer, TRAP primers and Taq polymerase and incubated at 30 °C for 30 min in a thermocycler followed by 3-step PCR at 94 °C /30 s, 59 °C/30 s, and 72 °C/1 min for 33 cycles. The PCR products were fractionated on nondenaturing 12.5% polyacrilamide gel and visualized by staining with ethidium bromide. The ladder of products with 6 base pair increment indicating telomerase activity was analyzed with NIH/Scion image analysis software. The cumulative band density for each lane was normalized to the corresponding band density of internal control (36 bp) and expressed as percent of the control. 

### 3.8. Western Blotting

Cell lysates were prepared in lysis buffer containing 1% Triton-X 100 (v/v), 10 mM Tris-HCl (pH 7.5), 5 mM EDTA, 150 mM NaCl, 10% glycerol, 2 mM sodium vanadate, 5 μg/mL leupeptin, 1 μg/mL aprotinin, 1 μg/mL pepstatinin, and 10 μg/mL 4–2-aminoethylbenzenesulfinyl fluoride). Lysates were clarified by centrifugation at 14,000 ×*g* for 10 min at 4 °C, and protein concentrations were determined by Bradford assay. Samples (75 μg) were boiled in an equal volume of sample buffer (20% glycerol, 4% SDS, 0.2% bromophenol blue, 125 mM Tris-HCl (pH 7.5), and 640 mM 2-mercaptoethanol) and separated on pre-casted Tris-glycine polyacrylamide gels using the XCell Surelock^TM^ Mini-Cell, in Tris-Glycine SDS running buffer, all from Novex (Invitrogen, Carlsbad, CA, USA). Proteins resolved on the gels were transferred to nitrocellulose membranes. Membranes were blocked with 5% milk in 10 mM Tris-HCl (pH 8.0); 150 mM NaCl with 0.05% Tween 20 (TPBS) and probed using target specific antibodies or β-actin as loading control and HRP-conjugated secondary antibody. Immune complexes were visualized with enhanced chemiluminescence. Protein bands were imaged and band densities analyzed using the NIH/Scion image analysis software. The protein band densities were normalized to the corresponding β-actin band densities. 

### 3.9. DNA Transfection

For overexpression of GPx or SOD-1, semi-confluent cultures MiaPaCa-2 and Panc-1 cells in 60 mm^2^ cell culture dishes were transfected with 2 µg of empty or expression plasmid DNA (pcDNA-GPx or pcDNA3-SOD-1 plasmids) using LipofectAMINE Plus reagent for 48 h. The expression level of GPx and SOD-1 was confirmed by immunoblotting. 

For silencing of Akt, cells were transfected with double stranded siRNA-Akt or non-targeting siRNA sequence using SignalSilence siRNA kit (Cell Signaling Technology, Beverly, MA, USA). Briefly, 2 × 10^6^ cancer cells were plated in 60 mm Petri dish for 24 h and treated with 3 mL of transfection medium containing 20 μg LipofectAMINE and 100 nM siRNA for 48 h. Akt expression was analyzed by immunoblotting.

### 3.10. Statistical Analysis

Most data are presented as means ± S.D. and outcomes for treated and untreated cells were compared by Student’s *t*-test. Differences were considered significant at *p* < 0.05.

## 4. Conclusions

This study provides evidence that inhibition of proliferation and induction of apoptosis in pancreatic cancer cells by CDDO-Me is mediated through a ROS-dependent pathway. Data also showed that ROS plays a crucial role in the attenuation of hTERT mRNA, hTERT telomerase activity and hTERT regulatory proteins (e.g., c-Myc, Sp1, NF-κB and p-Akt) by CDDO-Me as pretreatment with an antioxidant or overexpression of antioxidant enzymes (GPx and SOD-1) mitigated the antitelomerase activity of CDDO-Me. Thus, increased understanding of the mechanism of the antitelomerase activity of CDDO-Me would help in optimizing the therapeutic potential of CDDO-Me for pancreatic cancer. 
